# Supplementary material for: Single Cell Transcriptomic and Chromatin Profiles Suggest Layer Vb Is the Only Layer With Shared Excitatory Cell Types in the Medial and Lateral Entorhinal Cortex
Source: Front Neural Circuits. 2022 Jan 26;15:806154. doi: 10.3389/fncir.2021.806154 (PMC8826650; doi:10.3389/fncir.2021.806154)
Supplement: Supplementary file 14 [file Data_Sheet_1.ZIP › 4/knownResults.html]

4 - Homer Known Motif Enrichment Results


# Homer Known Motif Enrichment Results (4)

Homer *de novo* Motif Results  
Gene Ontology Enrichment Results  
Known Motif Enrichment Results (txt file)  
Total Target Sequences = 1000, Total Background Sequences = 46632

|  |  |  |  |  |  |  |  |  |  |  |  |
| --- | --- | --- | --- | --- | --- | --- | --- | --- | --- | --- | --- |
| Rank | Motif | Name | P-value | log P-pvalue | q-value (Benjamini) | # Target Sequences with Motif | % of Targets Sequences with Motif | # Background Sequences with Motif | % of Background Sequences with Motif | Motif File | SVG |
| 1 | T C G A A G T C C G T A A T C G A T G C C G A T A C T G A G T C A G C T A C T G | Tcf12(bHLH)/GM12878-Tcf12-ChIP-Seq(GSE32465)/Homer | 1e-41 | -9.668e+01 | 0.0000 | 292.0 | 29.20% | 5964.2 | 12.79% | motif file (matrix) | svg |
| 2 | C G A T T G C A T G C A G A T C C G T A A C T G T G A C G A C T C A T G A C T G | Tcf21(bHLH)/ArterySmoothMuscle-Tcf21-ChIP-Seq(GSE61369)/Homer | 1e-41 | -9.626e+01 | 0.0000 | 298.0 | 29.80% | 6175.6 | 13.24% | motif file (matrix) | svg |
| 3 | A T G C T A C G A G C T T G C A C G T A C G A T A C G T C T G A | DLX5(Homeobox)/BasalGanglia-Dlx5-ChIP-seq(GSE124936)/Homer | 1e-39 | -9.079e+01 | 0.0000 | 295.0 | 29.50% | 6255.5 | 13.41% | motif file (matrix) | svg |
| 4 | G T C A T G C A G C T A A G T C C G T A A C T G T G A C G C A T T C A G C A G T | Ap4(bHLH)/AML-Tfap4-ChIP-Seq(GSE45738)/Homer | 1e-38 | -8.840e+01 | 0.0000 | 350.0 | 35.00% | 8255.2 | 17.70% | motif file (matrix) | svg |
| 5 | C T A G T C G A T G A C A G T C C G T A A C T G G T A C A C G T A C T G A C T G | BHLHA15(bHLH)/NIH3T3-BHLHB8.HA-ChIP-Seq(GSE119782)/Homer | 1e-38 | -8.770e+01 | 0.0000 | 395.0 | 39.50% | 9948.0 | 21.33% | motif file (matrix) | svg |
| 6 | T G A C A G T C C G T A A C T G G T A C A C G T A C T G A C G T G A C T G A T C | Twist2(bHLH)/Myoblast-Twist2.Ty1-ChIP-Seq(GSE127998)/Homer | 1e-37 | -8.643e+01 | 0.0000 | 476.0 | 47.60% | 13195.9 | 28.29% | motif file (matrix) | svg |
| 7 | C T A G A G T C T A C G T A C G T G A C C G T A A C T G T A G C G C A T C A T G A T G C A G C T | Ascl1(bHLH)/NeuralTubes-Ascl1-ChIP-Seq(GSE55840)/Homer | 1e-37 | -8.600e+01 | 0.0000 | 401.0 | 40.10% | 10249.3 | 21.97% | motif file (matrix) | svg |
| 8 | T C A G C T A G A T G C G A C T T G C A C G T A A G C T A C G T C T G A T A C G | En1(Homeobox)/SUM149-EN1-ChIP-Seq(GSE120957)/Homer | 1e-36 | -8.444e+01 | 0.0000 | 506.0 | 50.60% | 14538.5 | 31.17% | motif file (matrix) | svg |
| 9 | T C G A T C G A A G T C C G T A C T A G T A G C A C G T A C T G | MyoG(bHLH)/C2C12-MyoG-ChIP-Seq(GSE36024)/Homer | 1e-35 | -8.260e+01 | 0.0000 | 300.0 | 30.00% | 6713.0 | 14.39% | motif file (matrix) | svg |
| 10 | T C G A C A G T A C T G A G C T C G T A C G T A A C G T A C G T C T G A T A G C | Dlx3(Homeobox)/Kerainocytes-Dlx3-ChIP-Seq(GSE89884)/Homer | 1e-33 | -7.822e+01 | 0.0000 | 254.0 | 25.40% | 5320.0 | 11.40% | motif file (matrix) | svg |
| 11 | A T C G A T G C A G T C T A G C G A C T T C G A G C T A G C A T A G C T C T G A | DLX1(Homeobox)/BasalGanglia-Dlx1-ChIP-seq(GSE124936)/Homer | 1e-33 | -7.799e+01 | 0.0000 | 410.0 | 41.00% | 10965.0 | 23.51% | motif file (matrix) | svg |
| 12 | A T C G A T C G A T G C G A C T T C G A C G T A G C A T A G C T C T G A T A C G | DLX2(Homeobox)/BasalGanglia-Dlx2-ChIP-seq(GSE124936)/Homer | 1e-33 | -7.742e+01 | 0.0000 | 442.0 | 44.20% | 12262.5 | 26.29% | motif file (matrix) | svg |
| 13 | T A C G T G C A A G T C C G T A A C G T T G A C A C G T A C T G A C T G G C A T | TCF4(bHLH)/SHSY5Y-TCF4-ChIP-Seq(GSE96915)/Homer | 1e-33 | -7.620e+01 | 0.0000 | 418.0 | 41.80% | 11365.3 | 24.36% | motif file (matrix) | svg |
| 14 | A G C T G T C A C G T A A C G T A C G T C T G A T C A G A T G C | Lhx2(Homeobox)/HFSC-Lhx2-ChIP-Seq(GSE48068)/Homer | 1e-32 | -7.485e+01 | 0.0000 | 343.0 | 34.30% | 8559.6 | 18.35% | motif file (matrix) | svg |
| 15 | A C T G C A G T A C G T C G T A C G T A A C G T A C T G C T G A | Nkx6.1(Homeobox)/Islet-Nkx6.1-ChIP-Seq(GSE40975)/Homer | 1e-30 | -6.983e+01 | 0.0000 | 616.0 | 61.60% | 20241.7 | 43.39% | motif file (matrix) | svg |
| 16 | T C A G A C G T T C G A T A G C A G T C C G T A A C T G G T A C A C G T A C T G A T C G A G T C | Atoh1(bHLH)/Cerebellum-Atoh1-ChIP-Seq(GSE22111)/Homer | 1e-30 | -6.951e+01 | 0.0000 | 314.0 | 31.40% | 7729.0 | 16.57% | motif file (matrix) | svg |
| 17 | A T G C T A C G A G T C G A C T T G C A C T G A G A C T A C G T C T G A T C A G | LHX9(Homeobox)/Hct116-LHX9.V5-ChIP-Seq(GSE116822)/Homer | 1e-30 | -6.934e+01 | 0.0000 | 403.0 | 40.30% | 11116.6 | 23.83% | motif file (matrix) | svg |
| 18 | T C G A T G A C G T A C C G T A C A G T T G A C A C G T A C T G A G C T A G C T | NeuroG2(bHLH)/Fibroblast-NeuroG2-ChIP-Seq(GSE75910)/Homer | 1e-28 | -6.615e+01 | 0.0000 | 402.0 | 40.20% | 11242.9 | 24.10% | motif file (matrix) | svg |
| 19 | C T G A T C A G G T A C G C T A A C T G T G A C G C A T C A T G | SCL(bHLH)/HPC7-Scl-ChIP-Seq(GSE13511)/Homer | 1e-27 | -6.305e+01 | 0.0000 | 787.0 | 78.70% | 29205.7 | 62.61% | motif file (matrix) | svg |
| 20 | C T G A T C A G A G T C C G T A A T C G A T G C C G A T A C T G A G T C G A C T A T C G A G T C | MyoD(bHLH)/Myotube-MyoD-ChIP-Seq(GSE21614)/Homer | 1e-26 | -6.131e+01 | 0.0000 | 227.0 | 22.70% | 5023.4 | 10.77% | motif file (matrix) | svg |
| 21 | A C G T T C G A T C G A A G T C G T C A T A C G A T G C A C G T A C T G A G C T | Myf5(bHLH)/GM-Myf5-ChIP-Seq(GSE24852)/Homer | 1e-24 | -5.733e+01 | 0.0000 | 210.0 | 21.00% | 4606.3 | 9.87% | motif file (matrix) | svg |
| 22 | A G C T A C G T A C T G A T G C A G T C C G T A C T G A T A C G | NF1-halfsite(CTF)/LNCaP-NF1-ChIP-Seq(Unpublished)/Homer | 1e-24 | -5.587e+01 | 0.0000 | 403.0 | 40.30% | 11855.6 | 25.41% | motif file (matrix) | svg |
| 23 | T A C G T A C G C T A G T C A G A G T C C G T A A T C G A T G C A C G T A C T G A G T C G A C T | Ascl2(bHLH)/ESC-Ascl2-ChIP-Seq(GSE97712)/Homer | 1e-22 | -5.185e+01 | 0.0000 | 273.0 | 27.30% | 7042.3 | 15.10% | motif file (matrix) | svg |
| 24 | T C G A A G T C C G T A A T C G T A G C A C G T A C T G A G C T A C G T A G T C | Ptf1a(bHLH)/Panc1-Ptf1a-ChIP-Seq(GSE47459)/Homer | 1e-22 | -5.156e+01 | 0.0000 | 533.0 | 53.30% | 17680.1 | 37.90% | motif file (matrix) | svg |
| 25 | C G T A C A T G A G T C G A C T T G C A C G T A A C G T A C G T C T G A T C A G | Lhx1(Homeobox)/EmbryoCarcinoma-Lhx1-ChIP-Seq(GSE70957)/Homer | 1e-20 | -4.779e+01 | 0.0000 | 322.0 | 32.20% | 9108.4 | 19.53% | motif file (matrix) | svg |
| 26 | T G A C A T G C C G T A A T C G A T G C C A G T C A T G A C T G A G T C G T A C | HEB(bHLH)/mES-Heb-ChIP-Seq(GSE53233)/Homer | 1e-20 | -4.654e+01 | 0.0000 | 392.0 | 39.20% | 11984.4 | 25.69% | motif file (matrix) | svg |
| 27 | G C A T C G T A C G A T A C T G A G T C G C T A C T G A C G T A C A G T A C T G C G T A T C A G | Oct6(POU,Homeobox)/NPC-Pou3f1-ChIP-Seq(GSE35496)/Homer | 1e-19 | -4.475e+01 | 0.0000 | 171.0 | 17.10% | 3795.9 | 8.14% | motif file (matrix) | svg |
| 28 | A C T G C A T G T C G A A C G T A C T G C G T A A T C G A C G T G T A C C G T A G A C T A G T C | Fos(bZIP)/TSC-Fos-ChIP-Seq(GSE110950)/Homer | 1e-18 | -4.218e+01 | 0.0000 | 157.0 | 15.70% | 3435.1 | 7.36% | motif file (matrix) | svg |
| 29 | G A C T C G T A A C G T A C T G A G T C C G T A C T G A C G T A C A G T A C G T G T C A T C A G | Brn1(POU,Homeobox)/NPC-Brn1-ChIP-Seq(GSE35496)/Homer | 1e-17 | -4.143e+01 | 0.0000 | 138.0 | 13.80% | 2855.8 | 6.12% | motif file (matrix) | svg |
| 30 | C G T A C G T A A G C T G A C T T G C A G T C A A C G T A G C T C T G A T C A G | Lhx3(Homeobox)/Neuron-Lhx3-ChIP-Seq(GSE31456)/Homer | 1e-17 | -4.138e+01 | 0.0000 | 419.0 | 41.90% | 13466.2 | 28.87% | motif file (matrix) | svg |
| 31 | C G T A T G A C T C G A A G T C C G T A A T C G A T G C A C G T A C T G A G T C | E2A(bHLH)/proBcell-E2A-ChIP-Seq(GSE21978)/Homer | 1e-17 | -4.131e+01 | 0.0000 | 310.0 | 31.00% | 9026.8 | 19.35% | motif file (matrix) | svg |
| 32 | G T A C G A C T C G T A C T G A T C G A C G T A G C T A C A G T C T G A T A C G | Mef2a(MADS)/HL1-Mef2a.biotin-ChIP-Seq(GSE21529)/Homer | 1e-17 | -4.044e+01 | 0.0000 | 137.0 | 13.70% | 2858.8 | 6.13% | motif file (matrix) | svg |
| 33 | C T G A T G A C T G A C C G T A A C G T T G A C A G C T C T A G A C G T G A C T | Olig2(bHLH)/Neuron-Olig2-ChIP-Seq(GSE30882)/Homer | 1e-17 | -3.951e+01 | 0.0000 | 441.0 | 44.10% | 14543.1 | 31.18% | motif file (matrix) | svg |
| 34 | T C A G T G A C G T A C C G T A A C G T T G A C A C G T T C A G A G C T G A C T | NeuroD1(bHLH)/Islet-NeuroD1-ChIP-Seq(GSE30298)/Homer | 1e-15 | -3.564e+01 | 0.0000 | 209.0 | 20.90% | 5530.5 | 11.86% | motif file (matrix) | svg |
| 35 | C A T G A G T C G A C T C G T A C G A T G C A T G A C T G C A T C G A T C T A G C A T G T G A C | Mef2b(MADS)/HEK293-Mef2b.V5-ChIP-Seq(GSE67450)/Homer | 1e-15 | -3.522e+01 | 0.0000 | 223.0 | 22.30% | 6067.0 | 13.01% | motif file (matrix) | svg |
| 36 | C A T G G T A C G A C T G C T A C G T A C G T A C G T A G C T A G A C T C T G A T C A G G T A C | Mef2c(MADS)/GM12878-Mef2c-ChIP-Seq(GSE32465)/Homer | 1e-15 | -3.486e+01 | 0.0000 | 141.0 | 14.10% | 3198.4 | 6.86% | motif file (matrix) | svg |
| 37 | C A T G A G T C A G C T C G T A C G A T C G A T G C A T G C A T C G A T C T G A C A T G T G A C | Mef2d(MADS)/Retina-Mef2d-ChIP-Seq(GSE61391)/Homer | 1e-14 | -3.427e+01 | 0.0000 | 77.0 | 7.70% | 1269.1 | 2.72% | motif file (matrix) | svg |
| 38 | C T A G T C G A A C G T A C T G C G T A A T G C A C G T G T A C C G T A A G C T G A T C G T A C | Atf3(bZIP)/GBM-ATF3-ChIP-Seq(GSE33912)/Homer | 1e-14 | -3.391e+01 | 0.0000 | 161.0 | 16.10% | 3914.3 | 8.39% | motif file (matrix) | svg |
| 39 | C T A G T A C G G A T C G T A C G C T A A G C T A G C T G T C A T C G A T A G C | Nanog(Homeobox)/mES-Nanog-ChIP-Seq(GSE11724)/Homer | 1e-14 | -3.355e+01 | 0.0000 | 748.0 | 74.80% | 29460.9 | 63.16% | motif file (matrix) | svg |
| 40 | A G T C G A C T C A G T A C T G C T A G T G A C G C T A A T G C G C A T A T C G C G A T A C T G G A T C G T A C G T C A C T G A | NF1(CTF)/LNCAP-NF1-ChIP-Seq(Unpublished)/Homer | 1e-14 | -3.311e+01 | 0.0000 | 108.0 | 10.80% | 2207.9 | 4.73% | motif file (matrix) | svg |
| 41 | T G C A C T G A A T G C G T C A A C G T A T G C A C G T A C T G A C T G T G C A | ZBTB18(Zf)/HEK293-ZBTB18.GFP-ChIP-Seq(GSE58341)/Homer | 1e-14 | -3.257e+01 | 0.0000 | 143.0 | 14.30% | 3361.3 | 7.21% | motif file (matrix) | svg |
| 42 | A C T G G T C A A C G T G C A T C G A T T C A G G T A C G T C A A C G T C T G A | Oct11(POU,Homeobox)/NCIH1048-POU2F3-ChIP-seq(GSE115123)/Homer | 1e-14 | -3.232e+01 | 0.0000 | 131.0 | 13.10% | 2972.3 | 6.37% | motif file (matrix) | svg |
| 43 | T C G A A C G T C A T G G C T A T A G C C G A T G T A C G C T A A C G T A T G C | AP-1(bZIP)/ThioMac-PU.1-ChIP-Seq(GSE21512)/Homer | 1e-13 | -3.119e+01 | 0.0000 | 175.0 | 17.50% | 4538.9 | 9.73% | motif file (matrix) | svg |
| 44 | C T A G T C G A G C A T C A T G G C T A T A G C C G A T G T A C C T G A A G C T | JunB(bZIP)/DendriticCells-Junb-ChIP-Seq(GSE36099)/Homer | 1e-13 | -3.056e+01 | 0.0000 | 136.0 | 13.60% | 3213.2 | 6.89% | motif file (matrix) | svg |
| 45 | A C T G C T A G T C G A C G A T C A T G G C T A A T C G C G A T G T A C G C T A A G C T G T A C | Fra1(bZIP)/BT549-Fra1-ChIP-Seq(GSE46166)/Homer | 1e-13 | -3.035e+01 | 0.0000 | 137.0 | 13.70% | 3256.2 | 6.98% | motif file (matrix) | svg |
| 46 | A T G C G A T C C G A T A C G T A C G T A C T G C A G T A G C T | Sox3(HMG)/NPC-Sox3-ChIP-Seq(GSE33059)/Homer | 1e-12 | -2.868e+01 | 0.0000 | 372.0 | 37.20% | 12481.9 | 26.76% | motif file (matrix) | svg |
| 47 | C G T A A C G T A G C T C G A T C T A G G T A C C G T A A G C T C G T A G C T A | Oct4(POU,Homeobox)/mES-Oct4-ChIP-Seq(GSE11431)/Homer | 1e-12 | -2.797e+01 | 0.0000 | 157.0 | 15.70% | 4066.0 | 8.72% | motif file (matrix) | svg |
| 48 | A T G C G T A C C G T A A G C T G C A T T A C G A G C T A G C T A G T C A G C T | Sox6(HMG)/Myotubes-Sox6-ChIP-Seq(GSE32627)/Homer | 1e-11 | -2.754e+01 | 0.0000 | 359.0 | 35.90% | 12031.9 | 25.79% | motif file (matrix) | svg |
| 49 | C A T G C T A G T C G A A C G T A C T G C G T A T A G C C G A T T G A C C G T A A G C T G A T C | Fra2(bZIP)/Striatum-Fra2-ChIP-Seq(GSE43429)/Homer | 1e-11 | -2.588e+01 | 0.0000 | 114.0 | 11.40% | 2681.9 | 5.75% | motif file (matrix) | svg |
| 50 | C A G T T G C A A C G T A C T G C G T A A T C G C G A T T G A C C G T A A C G T | BATF(bZIP)/Th17-BATF-ChIP-Seq(GSE39756)/Homer | 1e-10 | -2.440e+01 | 0.0000 | 151.0 | 15.10% | 4044.4 | 8.67% | motif file (matrix) | svg |
| 51 | A T G C A G T C G C A T A G C T A C G T T C A G C G A T A G C T G A T C A T C G | Sox10(HMG)/SciaticNerve-Sox3-ChIP-Seq(GSE35132)/Homer | 1e-10 | -2.334e+01 | 0.0000 | 343.0 | 34.30% | 11736.3 | 25.16% | motif file (matrix) | svg |
| 52 | G C T A G C A T G A C T G C A T T C A G G T A C G C T A G C A T C T G A G C T A T A G C G C T A C T G A C G A T C T A G | OCT4-SOX2-TCF-NANOG(POU,Homeobox,HMG)/mES-Oct4-ChIP-Seq(GSE11431)/Homer | 1e-9 | -2.206e+01 | 0.0000 | 74.0 | 7.40% | 1542.8 | 3.31% | motif file (matrix) | svg |
| 53 | C G T A A C G T A C T G G T A C C G T A A C G T C G T A C G T A A C G T A C T G A G T C C G T A A C G T C T G A G C A T | OCT:OCT-short(POU,Homeobox)/NPC-OCT6-ChIP-Seq(GSE43916)/Homer | 1e-9 | -2.144e+01 | 0.0000 | 191.0 | 19.10% | 5729.7 | 12.28% | motif file (matrix) | svg |
| 54 | G A C T C T A G A T G C A G T C G T C A T A C G A T G C A T C G | HIC1(Zf)/Treg-ZBTB29-ChIP-Seq(GSE99889)/Homer | 1e-9 | -2.082e+01 | 0.0000 | 379.0 | 37.90% | 13530.0 | 29.00% | motif file (matrix) | svg |
| 55 | A T G C G A T C C G T A A G C T C A G T A T C G G C A T A G C T G A C T A C T G | Sox17(HMG)/Endoderm-Sox17-ChIP-Seq(GSE61475)/Homer | 1e-8 | -2.053e+01 | 0.0000 | 180.0 | 18.00% | 5372.2 | 11.52% | motif file (matrix) | svg |
| 56 | A C G T A G T C A G T C C G A T A C G T A C G T A C T G A C G T A T G C G A C T A C T G T A C G | Sox21(HMG)/ESC-SOX21-ChIP-Seq(GSE110505)/Homer | 1e-8 | -1.989e+01 | 0.0000 | 374.0 | 37.40% | 13412.3 | 28.75% | motif file (matrix) | svg |
| 57 | A G T C A G T C C G A T A C G T A C G T A C T G A C G T A G C T A G T C A G T C | Sox4(HMG)/proB-Sox4-ChIP-Seq(GSE50066)/Homer | 1e-8 | -1.925e+01 | 0.0000 | 191.0 | 19.10% | 5893.6 | 12.63% | motif file (matrix) | svg |
| 58 | A G T C C G A T C T G A C G T A A C G T C A G T T C A G T G A C | Isl1(Homeobox)/Neuron-Isl1-ChIP-Seq(GSE31456)/Homer | 1e-8 | -1.921e+01 | 0.0000 | 389.0 | 38.90% | 14140.5 | 30.31% | motif file (matrix) | svg |
| 59 | G C T A A G C T T A C G G T C A C T G A C G A T C T G A G C A T C A G T A G T C | Brn2(POU,Homeobox)/NPC-Brn2-ChIP-Seq(GSE35496)/Homer | 1e-8 | -1.902e+01 | 0.0000 | 45.0 | 4.50% | 781.5 | 1.68% | motif file (matrix) | svg |
| 60 | C T A G T A C G G A T C G T C A T G C A A C G T T G C A G C T A T C G A T G C A | Hoxa9(Homeobox)/ChickenMSG-Hoxa9.Flag-ChIP-Seq(GSE86088)/Homer | 1e-8 | -1.869e+01 | 0.0000 | 534.0 | 53.40% | 20723.8 | 44.43% | motif file (matrix) | svg |
| 61 | A G C T C A T G G C A T G A T C T G C A C T A G G A T C A C G T | Tgif2(Homeobox)/mES-Tgif2-ChIP-Seq(GSE55404)/Homer | 1e-8 | -1.849e+01 | 0.0000 | 566.0 | 56.60% | 22235.0 | 47.67% | motif file (matrix) | svg |
| 62 | A T G C A G T C G A T C C G T A A C G T A C G T A C T G A C G T A G C T G A T C | Sox2(HMG)/mES-Sox2-ChIP-Seq(GSE11431)/Homer | 1e-7 | -1.800e+01 | 0.0000 | 202.0 | 20.20% | 6428.1 | 13.78% | motif file (matrix) | svg |
| 63 | C T G A T C G A C G T A A T G C C G T A C G T A C G A T C T A G T C A G G A T C | Sox15(HMG)/CPA-Sox15-ChIP-Seq(GSE62909)/Homer | 1e-7 | -1.759e+01 | 0.0000 | 235.0 | 23.50% | 7792.9 | 16.71% | motif file (matrix) | svg |
| 64 | G T C A G C A T G C T A C A G T C T A G G A T C C G T A C T G A C G T A C G A T | Oct2(POU,Homeobox)/Bcell-Oct2-ChIP-Seq(GSE21512)/Homer | 1e-7 | -1.706e+01 | 0.0000 | 109.0 | 10.90% | 2955.2 | 6.34% | motif file (matrix) | svg |
| 65 | A G T C C G A T A C T G A T C G T G A C G C T A C A T G A T C G T G A C C G A T A C T G T A G C G T A C G T C A | Tlx?(NR)/NPC-H3K4me1-ChIP-Seq(GSE16256)/Homer | 1e-6 | -1.584e+01 | 0.0000 | 104.0 | 10.40% | 2848.2 | 6.11% | motif file (matrix) | svg |
| 66 | C T A G T C G A C G A T A C T G C G T A T A C G A G C T T G A C G C T A A C G T G A T C T A G C | Fosl2(bZIP)/3T3L1-Fosl2-ChIP-Seq(GSE56872)/Homer | 1e-6 | -1.508e+01 | 0.0000 | 71.0 | 7.10% | 1733.2 | 3.72% | motif file (matrix) | svg |
| 67 | T G A C T A G C T C A G T C G A T C G A C G T A A G T C C G T A C G T A C G A T C T A G T A C G | Sox7(HMG)/ESC-Sox7-ChIP-Seq(GSE133899)/Homer | 1e-6 | -1.490e+01 | 0.0000 | 81.0 | 8.10% | 2085.2 | 4.47% | motif file (matrix) | svg |
| 68 | G A C T C G A T C T G A G T C A G A C T C G A T T C G A C G T A G C T A G C T A T G A C G T A C C G T A A C T G T G C A C G A T A C T G A C G T | Pitx1:Ebox(Homeobox,bHLH)/Hindlimb-Pitx1-ChIP-Seq(GSE41591)/Homer | 1e-6 | -1.428e+01 | 0.0000 | 61.0 | 6.10% | 1436.0 | 3.08% | motif file (matrix) | svg |
| 69 | C T A G T A C G G A T C G T A C C T G A A G C T T G C A G C T A C G T A G C A T G A T C G C T A | Hoxc9(Homeobox)/Ainv15-Hoxc9-ChIP-Seq(GSE21812)/Homer | 1e-5 | -1.370e+01 | 0.0000 | 129.0 | 12.90% | 3927.8 | 8.42% | motif file (matrix) | svg |
| 70 | C T A G T C G A A C G T A C T G C G T A T A G C C G A T G T A C C G T A A G C T G A T C G T A C | Jun-AP1(bZIP)/K562-cJun-ChIP-Seq(GSE31477)/Homer | 1e-5 | -1.349e+01 | 0.0000 | 54.0 | 5.40% | 1241.1 | 2.66% | motif file (matrix) | svg |
| 71 | G T C A A C G T C T A G G T C A C G T A G C A T C G T A C G T A G C A T C A G T A G T C C G T A C A G T C T A G C T G A | OCT:OCT(POU,Homeobox,IR1)/NPC-Brn2-ChIP-Seq(GSE35496)/Homer | 1e-5 | -1.336e+01 | 0.0000 | 15.0 | 1.50% | 151.7 | 0.33% | motif file (matrix) | svg |
| 72 | G C T A A C G T C T A G G T C A C G T A A C G T C G T A C G A T C A G T A G T C C G T A A C G T C T A G C T G A A T C G | OCT:OCT(POU,Homeobox)/NPC-Brn1-ChIP-Seq(GSE35496)/Homer | 1e-5 | -1.256e+01 | 0.0000 | 10.0 | 1.00% | 69.4 | 0.15% | motif file (matrix) | svg |
| 73 | G C T A C G A T C T A G G T A C G C T A C G A T C G T A G C T A C G A T C A G T G A T C G C T A | Pit1+1bp(Homeobox)/GCrat-Pit1-ChIP-Seq(GSE58009)/Homer | 1e-5 | -1.217e+01 | 0.0000 | 103.0 | 10.30% | 3055.8 | 6.55% | motif file (matrix) | svg |
| 74 | C T A G T A C G G A T C G T C A T C G A A C G T T C A G C G T A C G T A C G T A | Hoxd10(Homeobox)/ChickenMSG-Hoxd10.Flag-ChIP-Seq(GSE86088)/Homer | 1e-4 | -1.039e+01 | 0.0002 | 243.0 | 24.30% | 8919.6 | 19.12% | motif file (matrix) | svg |
| 75 | G C T A A C G T C T A G G T A C G C T A G A C T C T G A G C A T C A T G G A T C | Pit1(Homeobox)/GCrat-Pit1-ChIP-Seq(GSE58009)/Homer | 1e-4 | -1.039e+01 | 0.0002 | 250.0 | 25.00% | 9218.3 | 19.76% | motif file (matrix) | svg |
| 76 | C G A T T A C G T G A C G A C T C A T G C G T A T A C G A C G T G T A C C T G A | Bach2(bZIP)/OCILy7-Bach2-ChIP-Seq(GSE44420)/Homer | 1e-4 | -9.854e+00 | 0.0003 | 46.0 | 4.60% | 1142.9 | 2.45% | motif file (matrix) | svg |
| 77 | G A C T G T A C T G C A A C G T G A T C G C T A T C G A A C G T A G T C C G T A | Pdx1(Homeobox)/Islet-Pdx1-ChIP-Seq(SRA008281)/Homer | 1e-4 | -9.719e+00 | 0.0003 | 213.0 | 21.30% | 7735.6 | 16.58% | motif file (matrix) | svg |
| 78 | C T G A C G A T C T A G C G T A A G C T C G A T C A G T C T G A G A C T C T A G C T A G A T G C | PBX2(Homeobox)/K562-PBX2-ChIP-Seq(Encode)/Homer | 1e-4 | -9.223e+00 | 0.0006 | 182.0 | 18.20% | 6497.9 | 13.93% | motif file (matrix) | svg |
| 79 | T C G A A C G T A C G T C T G A G A T C T C A G G A C T G T C A C G T A A G C T G T C A C T A G A G C T A C G T T C G A | NFIL3(bZIP)/HepG2-NFIL3-ChIP-Seq(Encode)/Homer | 1e-3 | -8.946e+00 | 0.0007 | 128.0 | 12.80% | 4313.9 | 9.25% | motif file (matrix) | svg |
| 80 | T A C G C T G A C T A G T C G A C G T A A G T C C T G A A T C G G C A T T A G C G A C T A C T G A C G T A G C T A G T C G A C T | GRE(NR),IR3/A549-GR-ChIP-Seq(GSE32465)/Homer | 1e-3 | -8.573e+00 | 0.0010 | 34.0 | 3.40% | 801.5 | 1.72% | motif file (matrix) | svg |
| 81 | G A C T G C A T C T A G C G A T G A T C T C G A C A T G G A T C | Tgif1(Homeobox)/mES-Tgif1-ChIP-Seq(GSE55404)/Homer | 1e-3 | -8.067e+00 | 0.0017 | 510.0 | 51.00% | 21250.6 | 45.56% | motif file (matrix) | svg |
| 82 | C G T A C T G A C T A G C G T A C G T A A G T C C G T A C A G T G C A T G T C A C G A T A C T G A C G T G C A T G A T C | PGR(NR)/EndoStromal-PGR-ChIP-Seq(GSE69539)/Homer | 1e-3 | -7.746e+00 | 0.0023 | 54.0 | 5.40% | 1547.4 | 3.32% | motif file (matrix) | svg |
| 83 | C G T A C G A T G A C T G A C T T G A C C T G A A T G C C T G A T A G C A G T C A C G T T C G A C A T G T A C G G A C T A T C G G A C T A C G T C T G A T C G A C G T A | Brachyury(T-box)/Mesoendoderm-Brachyury-ChIP-exo(GSE54963)/Homer | 1e-3 | -7.605e+00 | 0.0026 | 60.0 | 6.00% | 1779.8 | 3.82% | motif file (matrix) | svg |
| 84 | T G A C C T G A C T A G C T G A C G T A A G T C C T G A A C G T G C A T T A G C G C A T A T C G G A C T G A C T G A T C | GRE(NR),IR3/RAW264.7-GRE-ChIP-Seq(Unpublished)/Homer | 1e-3 | -7.593e+00 | 0.0026 | 52.0 | 5.20% | 1484.4 | 3.18% | motif file (matrix) | svg |
| 85 | G A C T C T A G G A T C C A G T A C T G C T G A A T G C G C A T A T G C C T G A | MafA(bZIP)/Islet-MafA-ChIP-Seq(GSE30298)/Homer | 1e-3 | -7.226e+00 | 0.0038 | 165.0 | 16.50% | 6046.2 | 12.96% | motif file (matrix) | svg |
| 86 | C G T A A C G T A C G T A C G T A C G T A G T C A G T C C T G A A G C T A G C T | NFAT(RHD)/Jurkat-NFATC1-ChIP-Seq(Jolma\_et\_al.)/Homer | 1e-3 | -7.176e+00 | 0.0039 | 174.0 | 17.40% | 6433.5 | 13.79% | motif file (matrix) | svg |
| 87 | G T A C C G T A C G T A T A C G G C A T G T A C C G T A C A T G A G T C C G T A C G T A C G A T G C A T G C A T G A C T | MafF(bZIP)/HepG2-MafF-ChIP-Seq(GSE31477)/Homer | 1e-3 | -7.175e+00 | 0.0039 | 69.0 | 6.90% | 2154.5 | 4.62% | motif file (matrix) | svg |
| 88 | C G T A G A C T C G A T A T C G G T A C G C A T C A T G C G T A T A C G G C A T G T A C C G T A C A T G A T G C G C T A C T A G G C A T G C A T G C A T G A C T | MafB(bZIP)/BMM-Mafb-ChIP-Seq(GSE75722)/Homer | 1e-3 | -6.973e+00 | 0.0047 | 100.0 | 10.00% | 3395.4 | 7.28% | motif file (matrix) | svg |
| 89 | T A C G T C G A G A C T A C T G C T G A A G T C T C A G G A C T T G A C C T G A | Atf1(bZIP)/K562-ATF1-ChIP-Seq(GSE31477)/Homer | 1e-3 | -6.972e+00 | 0.0047 | 112.0 | 11.20% | 3881.0 | 8.32% | motif file (matrix) | svg |
| 90 | C T A G T C G A C G A T C T A G G C A T C A G T C T A G G A T C C G T A G T C A | CEBP:AP1(bZIP)/ThioMac-CEBPb-ChIP-Seq(GSE21512)/Homer | 1e-2 | -6.860e+00 | 0.0051 | 145.0 | 14.50% | 5257.8 | 11.27% | motif file (matrix) | svg |
| 91 | A G T C A T C G C T A G A G C T G A C T C T A G A G T C A G T C G C T A C A G T T C A G T C A G G A T C C T G A T C G A G A T C | RFX(HTH)/K562-RFX3-ChIP-Seq(SRA012198)/Homer | 1e-2 | -6.687e+00 | 0.0060 | 19.0 | 1.90% | 397.7 | 0.85% | motif file (matrix) | svg |
| 92 | C T A G A T G C A T G C C G A T A C T G G A C T A T G C G C T A T G A C A G C T T A G C G C T A | PBX1(Homeobox)/MCF7-PBX1-ChIP-Seq(GSE28007)/Homer | 1e-2 | -6.619e+00 | 0.0064 | 21.0 | 2.10% | 462.9 | 0.99% | motif file (matrix) | svg |
| 93 | G C T A A C T G T C G A C T G A C T G A A C G T T A G C C T G A C G T A C G A T | Cux2(Homeobox)/Liver-Cux2-ChIP-Seq(GSE35985)/Homer | 1e-2 | -6.362e+00 | 0.0082 | 103.0 | 10.30% | 3585.6 | 7.69% | motif file (matrix) | svg |
| 94 | A G T C G A T C G C T A C G A T A C G T T A C G G C A T C T G A G A C T A C T G A G T C G C T A C T G A T C G A C A G T | Oct4:Sox17(POU,Homeobox,HMG)/F9-Sox17-ChIP-Seq(GSE44553)/Homer | 1e-2 | -6.290e+00 | 0.0087 | 38.0 | 3.80% | 1060.7 | 2.27% | motif file (matrix) | svg |
| 95 | G C A T A T C G C A T G G T A C G C T A A G T C T C A G T G A C G T C A T G C A | Arnt:Ahr(bHLH)/MCF7-Arnt-ChIP-Seq(Lo\_et\_al.)/Homer | 1e-2 | -6.157e+00 | 0.0098 | 107.0 | 10.70% | 3773.4 | 8.09% | motif file (matrix) | svg |
| 96 | G T A C G C A T C T G A C G T A G A C T A G C T C A T G T G C A C T G A A C G T G A C T C G T A | Prop1(Homeobox)/GHFT1-PROP1.biotin-ChIP-Seq(GSE77302)/Homer | 1e-2 | -5.894e+00 | 0.0126 | 140.0 | 14.00% | 5182.5 | 11.11% | motif file (matrix) | svg |
| 97 | A C G T T G C A A G C T G A T C C T A G C T G A A G C T G T C A T C G A C G T A | CUX1(Homeobox)/K562-CUX1-ChIP-Seq(GSE92882)/Homer | 1e-2 | -5.871e+00 | 0.0128 | 152.0 | 15.20% | 5693.5 | 12.21% | motif file (matrix) | svg |
| 98 | T C A G G A C T C A G T C T G A A G C T C T A G G A C T T G C A C T G A A G T C | HLF(bZIP)/HSC-HLF.Flag-ChIP-Seq(GSE69817)/Homer | 1e-2 | -5.691e+00 | 0.0152 | 163.0 | 16.30% | 6190.7 | 13.27% | motif file (matrix) | svg |
| 99 | G A T C G C A T C T G A C G T A A G C T A G C T C G T A T C G A C T G A A C G T G C A T C G T A | Phox2a(Homeobox)/Neuron-Phox2a-ChIP-Seq(GSE31456)/Homer | 1e-2 | -5.481e+00 | 0.0185 | 89.0 | 8.90% | 3118.6 | 6.69% | motif file (matrix) | svg |
| 100 | C A T G C T A G A G C T G A C T C A T G A G T C G A T C G C T A C G A T C T A G T C A G G T A C C T G A T C G A | X-box(HTH)/NPC-H3K4me1-ChIP-Seq(GSE16256)/Homer | 1e-2 | -5.451e+00 | 0.0189 | 25.0 | 2.50% | 648.9 | 1.39% | motif file (matrix) | svg |
| 101 | T G C A C G T A G T C A A G C T A G T C G C T A T A G C C G A T C T A G G A T C | Gfi1b(Zf)/HPC7-Gfi1b-ChIP-Seq(GSE22178)/Homer | 1e-2 | -5.411e+00 | 0.0195 | 129.0 | 12.90% | 4789.3 | 10.27% | motif file (matrix) | svg |
| 102 | T C A G G T C A G C T A A G T C C G T A A T C G T C G A T C A G C G T A A C T G A C T G A C T G | ZNF768(Zf)/Rajj-ZNF768-ChIP-Seq(GSE111879)/Homer | 1e-2 | -5.410e+00 | 0.0195 | 10.0 | 1.00% | 171.2 | 0.37% | motif file (matrix) | svg |
| 103 | T C A G T A C G G A T C G T A C T C G A C G A T C T G A C G T A G C T A C G T A | Hoxd11(Homeobox)/ChickenMSG-Hoxd11.Flag-ChIP-Seq(GSE86088)/Homer | 1e-2 | -5.354e+00 | 0.0202 | 440.0 | 44.00% | 18619.5 | 39.91% | motif file (matrix) | svg |
| 104 | C T A G T A C G G A C T T G C A T G C A C G A T T A C G C T G A T C G A C T G A | Hoxa10(Homeobox)/ChickenMSG-Hoxa10.Flag-ChIP-Seq(GSE86088)/Homer | 1e-2 | -5.214e+00 | 0.0230 | 120.0 | 12.00% | 4439.3 | 9.52% | motif file (matrix) | svg |
| 105 | T C G A C T G A C T G A A T G C G T A C G T C A A G C T A G C T C G T A T C A G | Barx1(Homeobox)/Stomach-Barx1.3xFlag-ChIP-Seq(GSE69483)/Homer | 1e-2 | -4.836e+00 | 0.0333 | 107.0 | 10.70% | 3948.4 | 8.46% | motif file (matrix) | svg |
| 106 | T C G A G C A T A T G C C T G A A T G C T A G C A G T C G T A C T C G A A G C T | Srebp1a(bHLH)/HepG2-Srebp1a-ChIP-Seq(GSE31477)/Homer | 1e-2 | -4.776e+00 | 0.0350 | 35.0 | 3.50% | 1053.6 | 2.26% | motif file (matrix) | svg |
| 107 | A C T G T C A G A G C T G A C T C A T G A G T C A G T C G C T A C G A T C T A G T C A G G T A C C T G A T C G A | Rfx1(HTH)/NPC-H3K4me1-ChIP-Seq(GSE16256)/Homer | 1e-2 | -4.767e+00 | 0.0350 | 38.0 | 3.80% | 1167.9 | 2.50% | motif file (matrix) | svg |
| 108 | C G A T C T A G T C G A A G C T C G A T C T G A C G T A A G C T A C T G C T A G A T G C G A T C | Hoxb4(Homeobox)/ES-Hoxb4-ChIP-Seq(GSE34014)/Homer | 1e-2 | -4.747e+00 | 0.0353 | 47.0 | 4.70% | 1515.8 | 3.25% | motif file (matrix) | svg |
| 109 | C T A G C A G T C G T A A C G T A G T C A C T G C G T A A G C T A G T C G A T C | HNF6(Homeobox)/Liver-Hnf6-ChIP-Seq(ERP000394)/Homer | 1e-2 | -4.682e+00 | 0.0374 | 116.0 | 11.60% | 4350.0 | 9.33% | motif file (matrix) | svg |
| 110 | A G C T G C A T A C T G A C G T A G T C A C G T C T A G T A C G | Smad3(MAD)/NPC-Smad3-ChIP-Seq(GSE36673)/Homer | 1e-2 | -4.675e+00 | 0.0374 | 461.0 | 46.10% | 19762.0 | 42.36% | motif file (matrix) | svg |
